# Guided imagery relaxation therapy on preoperative anxiety: a randomized clinical trial*

**DOI:** 10.1590/1518-8345.2850.3101

**Published:** 2018-11-29

**Authors:** Márcia Marques dos Santos Felix, Maria Beatriz Guimarães Ferreira, Lucas Felix de Oliveira, Elizabeth Barichello, Patricia da Silva Pires, Maria Helena Barbosa

**Affiliations:** 1 Universidade Federal do Triângulo Mineiro, Departamento Didático Científico de Enfermagem na Assistência Hospitalar, Uberaba, MG, Brasil.; 2 Prefeitura Municipal de Uberaba, Secretaria de Saúde, Uberaba, MG, Brasil.; 3 Universidade Federal da Bahia, Instituto Multidisciplinar em Saúde, Vitória da Conquista, BA, Brasil.

**Keywords:** Imagery (Psychotherapy, Relaxation, Anxiety, Hydrocortisone, Clinical Trial, Bariatric Surgery

## Abstract

**Objective:**

to evaluate the effect of relaxation therapy with guided imagery on state anxiety and cortisol in the immediate preoperative period in patients submitted to bariatric surgery by videolaparoscopy.

**Method:**

a randomized, triple-blind clinical trial in a large teaching hospital in the interior of Minas Gerais. Twenty-four patients who would undergo video-laparoscopic bariatric surgery were randomly allocated in two groups, namely 12 in the control group and 12 in the experimental group. State anxiety was assessed by the State-Trait Anxiety Inventory, and blood cortisol levels were measured before and after the intervention or standard care. Descriptive analyzes were used for the quantitative variables and Student’s t-test for independent samples, in the analysis of the differences between the state anxiety scores and cortisol levels.

**Results:**

the experimental group presented a statistically significant reduction of the state anxiety scores (p = 0.005) as well as of cortisol levels (p <0.001) after the intervention.

**Conclusion:**

guided imagery relaxation therapy is an effective nursing intervention for the reduction of state anxiety and blood cortisol levels in the preoperative period in patients undergoing video-laparoscopic bariatric surgery. Brazilian Registry of Clinical Trials: RBR-5qywrf.

## Introduction

Anxiety can be defined as an unpleasant emotional state involving feelings of apprehension and nervousness, being known to cause abnormal hemodynamics as a consequence of sympathetic, parasympathetic and endocrine stimulation^(^ ^1^ ^)^. Most patients awaiting elective surgery experience anxiety, since the preoperative phase is considered the period when the patient is most vulnerable, becoming prone to emotional imbalances^(^ ^1^ ^-^ ^2^ ^)^.

The incidence of preoperative anxiety varies according to the surgery scenario, ranging from 40 to 76%. High levels are associated to large elective surgical procedures^(^ ^3^ ^-^ ^5^ ^)^.

Bariatric surgery, a large elective procedure for morbidly obese individuals, results, in the long term, in weight loss, improvement or resolution of comorbidities, better quality of life and greater survival. This surgery is indicated for adults with Body Mass Index (BMI) ≥ 35 kg/m^2^, with one or more significant obesity-related comorbidities^(^ ^6^ ^)^. The lack of guidance about the surgery and an adequate therapeutic relationship with the patient by the health team can cause a state of anxiety and depression throughout the hospitalization period^(^ ^1^ ^)^.

Anxiety is recognized by patients for subjective aspects related to psychological issues, such as reports of inability to relax, insomnia, irritability and impatience, which is most often identified by nurses, but few include it in the systematization of their care or register alternatives to minimize it^(^ ^7^ ^)^.

In response to an acute stressor, such as preoperative anxiety, the hypothalamus secretes corticotrophin-releasing hormone (CRH), which travels to the anterior pituitary gland and stimulates the secretion of adrenocorticotrophic hormone (ACTH), which in turn is released in the blood flow and eventually reaches the adrenal cortex, where it stimulates the release of cortisol^(^ ^8^ ^-^ ^9^ ^)^.

Cortisol, then released from preoperative anxiety, is the major adrenal glucocorticoid and plays a central role in metabolism in the body’s response to stress; it reduces inflammation, promotes analgesia, contributes to the functioning of the immune system and maintains constant levels of blood sugar, as well as blood pressure^(^ ^8^ ^-^ ^9^ ^)^.

Therefore, adequate management of preoperative anxiety may result in improved outcome of surgery, greater patient satisfaction, and decreased hospital costs^(^ ^5^ ^,^ ^10^ ^)^. Several mind-body approaches can help alleviate the anxiety patients experience before or during stressful situations, such as elective surgical procedures^(^ ^2^ ^)^. Promising approaches include meditative practices and relaxation techniques associated with guided imagery.

Guided imagery is a mind-body intervention that uses the patient’s own imagination and mental processing to form a mental representation of an object, place, event, or situation perceived through the senses. It is considered a relaxation technique that focuses on the interaction between brain, mind, body and behavior. The patient is instructed to focus on pleasing images to replace negative or stressful feelings. Guided imagery can be self-directed, conducted by a professional or by a recording^(^ ^11^ ^-^ ^12^ ^)^.

The present study was porposed considering the need of evidence for the use of guided imagery relaxation therapy intervention in the context of nursing care. It is believed that given the mind-body connection between preoperative anxiety and large elective surgical procedures, this intervention might be effective in reducing preoperative anxiety in patients undergoing bariatric surgery.

In view of the above, this study aimed to evaluate the effect of guided imagery relaxation therapy on state anxiety and cortisol in the immediate preoperative period in patients submitted to bariatric surgery by videolaparoscopy.

## Method

This research was performed according to the recommendations of the Consolidated Standards of Reporting Trials (CONSORT) for trials evaluating non-pharmacological treatments^(^ ^13^ ^-^ ^14^ ^)^.

This is a triple-blind, parallel, randomized clinical trial consisting of two groups, namely the experimental group (EG), consisting of participants who received a guided imagery session associated with relaxation, and the control group (CG), composed of participants who received standard care. The study was developed from February 2016 to October 2017, in a large teaching hospital in the interior of Minas Gerais state.

A list of patients who had already been prepared by the surgical team and were waiting for the surgery (n = 53) were used as the population of this investigation. The number of participants was n = 24, 12 in the EG and 12 in the CG. In the topic “results”, the power analysis will be presented for this sample size for the main outcome (anxiety levels).

The inclusion criteria were being submitted to bariatric surgery by videolaparoscopy and being 18 years of age or older. Exclusion criteria were hearing loss or deficits.

The evaluated intervention was a complementary guided imagery relaxation therapy, based on Guided Meditation for Procedures or Surgery, created by Tusek^(^ ^15^ ^)^, and in the concept of guided imagery relaxation therapy, described by Fitzgerald and Langevin^(^ ^11^ ^)^.

The intervention was developed by one of the researchers with the collaboration of a psychologist and tested in a pilot study, with five patients undergoing a major surgery, which were not included in the final sample. The EG participants received the intervention in the immediate preoperative period (up to 24 hours before the surgery), performed by one of the researchers, trained to use this technique.

The therapy was conducted by using the Multilaser brand Headset Gamer PH073 headphones and connected to the You Sound brand MP3 player, with a 20-minute audio recording. International studies indicate that the relaxation therapy session with guided imagery, lasting 18 to 20 minutes, has physiological effects on the immune system and stress levels, leading to a decrease in anxiety^(^ ^16^ ^-^ ^18^ ^)^.

Guided imagery therapy can incorporate the use of relaxation techniques, such as diaphragmatic breathing and musical background, to help the participant focus and stay focused^(^ ^11^ ^,^ ^15^ ^)^. In this study, the therapy session started with a soft background music, with nature sounds (sea and seagulls) and an audio that invited the participant to stand in a comfortable position in the bed with their eyes closed and then to perform movements of slow and expansive breathing and relaxation in various parts of the body.

Scenes commonly used to induce relaxation in guided imagery therapy include watching a sunset or moonlight, sitting on a warm beach, or floating through water or space^(^ ^11^ ^)^. In this work, the audio led the participants to imagine themselves on a beach where they walked barefoot on the soft sand, then directed them to lie on the warm and soft sand, listening to the noise of the sea, and continued to guide them to feel completely well, in peace, without worries, anxiety, tensions, anguish and pain. To conclude, the participants were directed to open their eyes very slowly and to be comfortable.

Participants of the CG received standard care in the immediate preoperative period (up to 24 hours before surgery). Standard care consisted of resting in the bed and using earphones with no audio, connected to an MP3 player, for 20 minutes.

The primary outcome of the research was the reduction of preoperative anxiety scores assessed by the State-Trait Anxiety Inventory (STAI) in the immediate preoperative period (up to 24 hours before surgery), before and after the application of the relaxation therapy with guided imagery. The secondary outcome was the reduction of preoperative blood cortisol levels, measured by venipuncture, before and after the application of guided imagery relaxation therapy and later determined by means of the electrochemiluminescence immunoassay (ECLIA) method.

The randomization process was performed with the aid of a randomization scheme generated by the Randomization.com website. The technique used was randomization in blocks of ten. Four blocks with 10 randomized participants were generated in each block. This procedure was performed by a statistician without clinical involvement in the trial. After the generation of the random sequence, a list was generated, numbered sequentially for the allocation of patients to the groups. The study participants, the researcher who applied the data collection instrument and the laboratory technicians who performed the cortisol dosage were blinded as to the type of intervention that each participant received, which characterizes this study as triple-blind.

For the data collection, an instrument was developed for this study, submitted to face validity by three specialists in the addressed issue. This instrument consisted of participant’s identification data (name, bed, medical chart number), sociodemographic variables (date of birth, age, sex, profession and years of study) and clinical variables (surgery performed and comorbidities). To measure anxiety levels, the State-Trait Anxiety Inventory (STAI) was used, an instrument translated and validated for the Portuguese language in 1979^(^ ^19^ ^)^. The STAI is composed of two distinct self-assessment subscales, designed to measure two distinct anxiety concepts: state and trait. Both subscales consist of statements whose intensity of responses varies from 1 to 4 points, and the total score can vary from 20 (minimum) to 80 (maximum). The trait-anxiety subscale requires the participant to describe how he or she generally feels, whereas the subscale of state-anxiety requires the participant to indicate how he or she feels at a given time or situation. Blood cortisol levels were measured before and after the intervention or standard care.

For the determination of cortisol levels, blood samples (3 ml) were obtained by venipuncture with aseptic technique and disposable material; and then placed in tubes containing separator gel and centrifuged at 3000 rpm for 10 minutes for plasma extraction. The decanted plasma was placed in a cryotube labeled with each patient’s code and frozen at 80° C for further processing. The cortisol dosage was performed by the Clinical Analyzes and Pathology Anatomy Laboratory Unit of the study’s host institution, determined by the electrochemiluminescence immunoassay (ECLIA) method, in a Roche Cobas^®^ E601 immunoassay analyzer.

For the accomplishment of the collection, the research team was composed of three female nurses/researchers and PhD students. The distribution of the research team was as follows: two researchers were responsible for collecting the blood samples and applying the data collection instrument before and after the intervention, and one researcher applied the intervention or standard care. In addition to the data collection team, the study was attended by a psychologist who assisted in the elaboration of the intervention and training of the researcher responsible for the application thereof.

The data collection occurred after participants were allocated through the randomization list, when they were already hospitalized for the surgery. The collection was made in the participant’s bed once a day in the immediate preoperative period (up to 24 hours before surgery). Participant identification data were collected and then the State-Trait Anxiety Inventory was applied. Subsequently, blood was collected for analysis of cortisol levels and then the relaxation therapy applied, with guided imagery for the EG and standard care for the CG. After the intervention or standard care, the Anxiety Inventory was again applied (subscale for state-anxiety only) and another blood sample was collected for analysis of cortisol levels.

**Figure 1:**
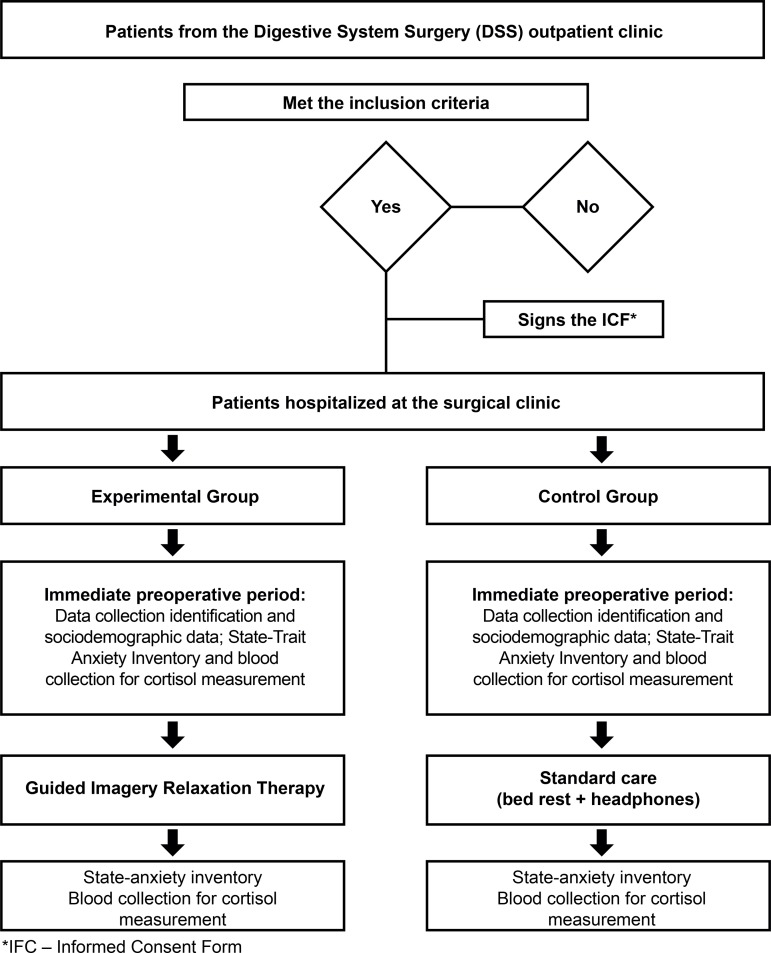
Data collection procedure. Uberaba, MG, Brazil, 2017

The scheme used for the data collection procedure is represented in Figure 1.

The double typing technique was used, and the collected data were analyzed through the Statistical Package Social Science (SPSS 21.0) software. The level of significance was α = 0.05.

To test the hypothesis of homogeneity of the two groups (CG and EG), the t-test was used for independent samples in the quantitative variables (age and trait-anxiety) and the chi-square test of homogeneity for the gender categorical variable.

Continuous variables were submitted to a normality test using the Shapiro-Wilk test; descriptive statistics were used for quantitative variables, through descriptive measures of centrality and dispersion; paired t-test for the analysis of the differences between the state-anxiety scores and cortisol levels before and after the intervention or intra-group standard care; Student’s t-test for independent samples to analyze the mean difference between anxiety scores before and anfer the intervention or standard care among groups to assess the effect of guided imagery relaxation therapy on state anxiety and cortisol levels.

To meet the ethical criteria, the anonymity of the participants was maintained and the Informed Consent Form (ICF) was signed. This study was approved by the Research Ethics Committee of the Federal University of Triângulo Mineiro, Approval Ceryificate (CAAE) number 40750114.3.0000.5154, opinion number 975.447/2015, and registered in the database of Brazilian Registry of Clinical Trials (REBEC), with primary identifier RBR-5qywrf.

## Results

**Figure 2:**
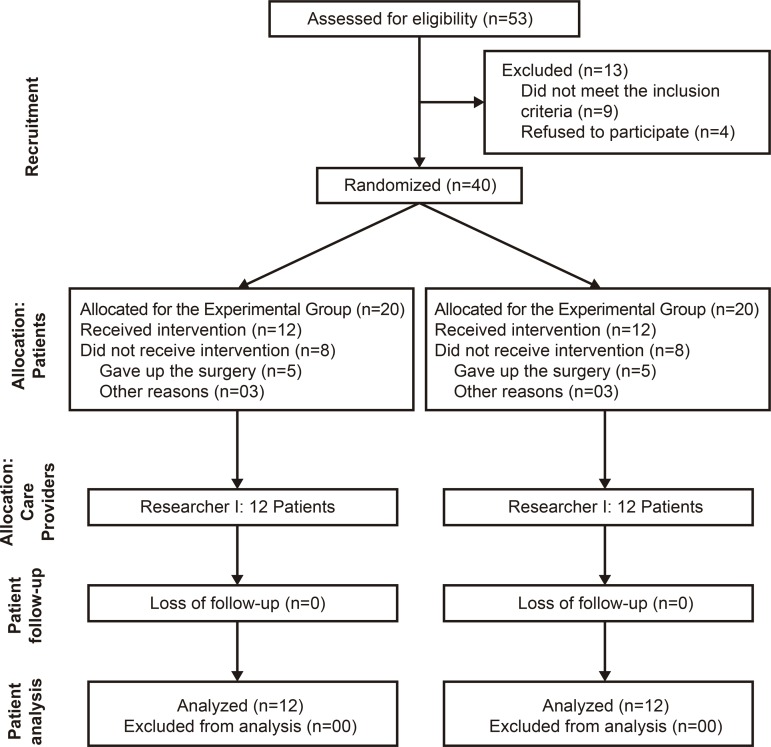
Flowchart of the participants involved in the study. Uberaba, MG, Brazil, 2017

The eligible population was 53 participants, and 24 concluded the study, with a loss of 29 (54.72%) patients (Figure 2).

After randomization, eight participants in the experimental group did not receive the intervention: four gave up surgery because they had lost weight with diets and exercises proposed by the bariatric team; one gave up because she became pregnant; two had not been submitted to surgery until the end of the data collection period; and one was prevented from undergoing the surgery because he presented severe psychological problems. In the control group, eight participants did not receive the intervention: four gave up surgery because they had lost weight with diets and exercises proposed by the bariatric team; one gave up because he moved to another state; and three underwent laparotomy surgery because of adhesions from previous surgeries that did not allow the accomplishment of bariatric surgery by videolaparoscopy.

The study sample consisted of 24 participants, 12 in the control group (CG) and 12 in the experimental group (EG). Table 1 shows the characterization data of the sample and the homogeneity tests.

**Table 1:** Sociodemographic and clinical characterization and homogeneity test for the variables age, state-anxiety and sex, considering the control and experimental groups. Uberaba, MG, Brazil, 2017

Sociodemographic and clinical variables	CG* (n=12)		EG^†^ (n=12)		p^§^

	Mean	SD^‡^	Mean	SD^‡^	
Age in years	46.50	8.82	37.92	9.69	0.033^||^
Trait-anxiety	44.42	5.03	45.92	5.90	0.510^||^
Sex	n	%	n	%	
Female	12	100	10	83.30	0.140^¶^
Male	00	00	02	16.70	
Profession	n	%	n	%	
Housewife	08	66.70	04	33.30	-
Hairdresser	01	08.30	01	8.30	
Teacher	00	00	02	16.70	
General assistant	00	00	02	16.70	
Other	03	25.00	03	25.00	
Level of schooling	n	%	n	%	
Elementary School	09	75.00	07	58.30	-
High school	01	08.30	03	25.00	
Higher education	02	16.70	02	16.70	
Comorbidities	n	%	n	%	
No comorbidity	01	8.30	02	16.70	-
T2DM**	03	25.00	01	8.30	
AH^††^	01	8.30	05	41.70	
T2DM** +AH^††^	05	41.70	03	25.00	
T2DM** +AH^††^ + Bronchitis	00	00	01	8.30	
T2DM** +AH^††^ + Arthrosis	01	8.30	00	00	
T2DM** +AH^††^ + Hypothyroidism	01	8.30	00	00	
Surgery	n	%	n	%	
Laparoscopic Gastric Bypass	12	100	12	100	-

*CG – Control Group; †EG - Experimental Group; ‡SD - Standard deviation; §p – p-value; || - Homogeneity test (p-value) for the calculation of the t-test; ¶ - Homogeneity test (p-value) for the Chi-Square test; **T2DM - Type II Diabetes Mellitus; ††AH - Arterial hypertension.

The homogeneity of the study sample was investigated for age, trait-anxiety levels (t-test) and sex (Chi-Square test). The tests showed that the control and experimental groups are comparable, considering these variables.

Of the participants, 22 (91.7%) were female, 12 (50%) were housewives and 16 (66.70%) had finished elementary school. The mean age was 42.21 years (SD = 10.06) and the average schooling was 8.88 (SD = 2.32) years of study. Regarding the clinical data collected in the participants’ charts, eight (33.3%) had arterial hypertension associated with type II diabetes mellitus, whereas another six (25%) had only arterial hypertension. All participants were submitted to the same surgical technique, laparoscopic gastric bypass.

The results of the intragroup analysis, with measures of central tendency, variability and statistical significance for the state-anxiety scores according to the State-Trait Anxiety Inventory (STAI) and cortisol levels before and after intervention or standard care, considering the control and experimental groups in the immediate preoperative period, demonstrated that there was a decrease in state-anxiety and cortisol levels, both for the control group and for the experimental group (Table 2).

**Table 2:** Measures of central tendency, variability and statistical significance of the change in state-anxiety scores, according to the State-Trait Anxiety Inventory (STAI) and cortisol levels before and after the intervention, considering the control and experimental groups, in the immediate preoperative period. Uberaba, MG, Brazil, 2017

Parameter	Pre-intervention		Post-intervention		p^†^

	Mean	SD*	Mean	SD*	
Level of state-anxiety					
Control Group (n^‡^=12)	47.67	3.82	46.83	3.76	0.005
Experimental Group (n^‡^=12)	47.50	2.61	43.00	3.54	0.001
Cortisol level					
Control Group (n^‡^=12)	8.61	5.43	8.16	5.29	0.001
Experimental Group (n^‡^=12)	9.87	6.10	7.95	5.37	<0.001

*SD - Standard deviation; †p - p-value for the calculation of the paired t-test; ‡n - number of participants

In investigating the efficacy of guided imagery relaxation therapy on the mean of the difference (reduction) between state-anxiety scores and cortisol levels (intergroup analysis) before and after the intervention, the results showed that the reduction in levels of state-anxiety was higher in the experimental group, with a statistically significant difference (p = 0.005). Regarding cortisol levels, the reduction was also higher in the experimental group, with a statistically significant difference (p <0.001) (Table 3).

**Table 3:** Mean of the difference (reduction) between the state-anxiety and cortisol levels before and after the intervention, considering the control and experimental groups, in the preoperative period. Uberaba, MG, Brazil, 2017

Parameter	n*	Mean	SD^†^	p^‡^
State-anxiety				
Control Group	12	0.83	0.83	0.005
Experimental Group	12	4.50	3.65	
Cortisol				
Control Group	12	0.45	0.37	<0.001
Experimental Group	12	1,92	0,90	

*n - number of participants; †SD - Standard deviation; ‡p - p-value referring for the calculation of the Student’s t-test for independent samples

The power analysis, considering the sample size of n = 24, the significance level α = 0.05 and the data in Table 3 (Mean of the difference between the pre- and post-intervention state-anxiety scores in the immediate preoperative period), for the control and experimental groups, revealed that the a priori statistical power reached was 99%.

## Discussion

The findings from the present study showed that the intervention reduced anxiety and cortisol levels in the immediate preoperative period, corroborating evidence available in the literature that several non-pharmacological approaches are available to reduce these symptoms. Using a principle-based concept analysis to examine the state of science, researchers analyzed 12 studies to clarify the concept of anxiety relief using complementary therapies in the perioperative period, and observed that complementary therapies (acupuncture, music, guided imagery, essential oils and relaxation) associated with conventional medical treatment may be effective in the perioperative period and produce substantial benefits for surgical patients^(^ ^20^ ^)^.

Preoperative anxiety is associated with problems such as difficult venous access, demand of higher doses of anesthetic agents and analgesics, and contribute to postoperative complications^(^ ^4^ ^-^ ^5^ ^,^ ^21^ ^)^. High levels of anxiety negatively influence the physiological parameters and interfere with the postoperative period, which can lead to increased hospitalization time^(^ ^4^ ^,^ ^10^ ^)^.

In a study of 52 inpatients and outpatients, prior to cardiac catheterization, the researchers evaluated the effectiveness of massage with or without guided imagery in reducing anxiety. Participants in the experimental group (n = 28) received a Swedish technique massage (on the back, scalp, arms and feet) or guided imagery (headphone and a 20-minute relaxation CD with soft background music, progressive relaxation, followed by relaxing suggestions, guiding the patient to a cozy and beautiful beach scene) together with massage (n = 24) before cardiac catheterization. A comparison group (corresponding in relation to age, sex, procedure, and status as inpatient or outpatient) was retrospectively selected from a list of patients who received cardiac catheterization during the same period as the treatment group, but they did not receive massages or guided imagery. A 10-point analogue scale was used to assess anxiety levels. Blood pressure and heart rate were measured before and after intervention in the participants and in the comparison group. The authors observed that both massage and guided imagery combined with massage showed significant reductions in self-reported anxiety, and participants who received the intervention had lower blood pressure and heart rate versus the comparison group^(^ ^22^ ^)^.

Complementary therapies have a positive impact in reducing the patient’s stress and suffering, since their effects reduce the activity of the autonomous nervous system, which is responsible for the control of visceral and homeostatic functions^(^ ^23^ ^)^. However, no studies were found that evaluated the efficacy of guided imagery relaxation therapy in the reduction of preoperative anxiety and blood cortisol.

The literature has demonstrated that, in addition to pharmacological treatments, other types of complementary interventions have a positive effect on reducing preoperative anxiety. In order to examine how mood music affects the level of state-anxiety and vital signs in patients scheduled for elective surgeries, researchers evaluated 159 participants divided into intervention group (n = 82) and control group (n = 77). Eighty-two interviewees were submitted to mood music; of these, 42 listened to classical music and 40 to New Age music. Seventy-seven participants heard no music. Data on anxiety were collected before and after the intervention, using the State-Trait Anxiety Inventory, a Visual Analogue Scale, and vital signs measurement. The authors observed that listening to mood music was associated with lower levels of state-anxiety and normalization of vital signs^(^ ^24^ ^)^.

Exploring the impact of timed and self-selected music as a safe and noninvasive intervention in reducing preoperative anxiety was the goal of a randomized clinical trial in which the researchers evaluated 133 patients admitted for surgery. Participants were randomized to the 30-minute (group A, n = 41) or 15-minute (group B, n = 47) intervention groups or to the control group (group C, n = 45). Participants in the experimental groups selected and listened to one of four musical genres: classic, jazz, religious or nature sounds, whereas the control group received only standard care that did not include music. Data were collected using the State-Trait Anxiety Inventory and a Visual Analog Scale. The study results showed that state-anxiety was lower after participants listened to 15 or 30 minutes of music, with statistically significant differences^(^ ^2^ ^)^.

The neurochemical changes induced by mind-body interventions can produce an anxiolytic effect. In a study aimed at evaluating the modulation of perioperative anxiety by a mind-body intervention technique called Raja Yoga meditation, the researchers evaluated 150 patients undergoing elective coronary artery bypass grafting using a randomized clinical trial. Participants were randomly assigned to Raja Yoga group and control group (guidelines on surgery and anesthesia). Anxiety was measured using a visual analogue scale before the beginning of the intervention or guidance to the participants (T1), in the morning of the day of surgery (T2), in the second postoperative day (T3) and in the fifth postoperative day (T4). Serum cortisol level was measured in the morning of the day of surgery (T1), in the second postoperative day (T2) and in the fifth postoperative day (T3), respectively. It was found that the participants’ level of anxiety before surgery (T1) and on the day of surgery (T2) was comparable between the two groups. However, in the second postoperative day (T3), participants submitted to Raja Yoga sessions presented lower level of anxiety compared to the control group, and on the fifth postoperative day (T4), it was observed that the practice of Raja Yoga resulted in significant decline in blood cortisol levels and anxiety levels^(^ ^25^ ^)^.

Reducing preoperative anxiety may improve surgical outcome, decrease length of hospital stay, minimize post-operative disruption, and increase overall patient satisfaction with perioperative care^(^ ^10^ ^)^. In this sense, it is important to use complementary therapies, such as guided imagery relaxation therapy, to reduce anxiety and consequently offer a better quality of life to the patient during the treatment process.

Participant losses after randomization can be considered as a limitation of the study. There were eight losses in the experimental group and eight in the control group. These losses occurred due to the non-performance of the surgery: ten participants gave up performing the procedure, one could not undergo surgery due to psychological problems, three had undergone open surgeries and two had not yet undergone surgery due to problems at the study institution, which caused a reduced sample.

The relaxation techniques and guided imagery, used as a strategy in the nursing intervention, fit within the integrative and complementary practices, contributing to broaden the field of action of the nurse for a quality care, promoting a more effective way to face stressful situations, as well as bringing comfort and well-being to the patient during the perioperative period^(^ ^26^ ^)^. Few studies have been conducted to investigate the efficacy of this technique in reducing preoperative anxiety and blood cortisol levels. For this reason, other investigations are necessary on this subject in order to establish new data, as well as new procedures in the patient care in the preoperative period of elective surgeries, aiming at minimizing physical, psychic and spiritual suffering, thus providing a more humanized care.

## Conclusions

This study allowed concluding that the investigated intervention proved to be effective, evidencing statistically significant differences between the groups in the levels of state-anxiety (p = 0.005) and blood cortisol concentration (p <0.001) in the immediate preoperative period, in patients submitted to videolaparoscopic bariatric surgery.

The present study contributed with important evidence related to the effect of relaxation therapy with guided imagery on preoperative anxiety and cortisol in patients submitted to a large surgery by videolaparoscopy. However, for the generalization of these results, future research is needed to evaluate the effect of this intervention on blood cortisol levels. Further studies with larger samples may confirm these results and provide additional information.
